# Supplementary material for: In Vitro Cytotoxicity of Antiresorptive and Antiangiogenic Compounds on Oral Tissues Contributing to MRONJ: Systematic Review
Source: Biomolecules. 2023 Jun 10;13(6):973. doi: 10.3390/biom13060973 (PMC10296421; doi:10.3390/biom13060973)
Supplement: Supplementary file 1 [file biomolecules-13-00973-s001.zip › biomolecules-2399970 - Supplementary File 1.pdf]

Supplementary File 1

Table S1: Full OHAT Risk of Bias Analysis

Ref	Was administered dose or exposure level adequately randomised?	Was allocation to study groups adequately concealed?	Were experimental conditions identical across study groups?	Were the research personnel and human subjects blinded to the study group during the study?	Were outcome data complete without attrition or exclusion from analysis?	Can we be confident in the exposure characterization?	Can we be confident in the outcome assessment?	Were all measured outcomes reported?	Were statistical methods appropriate?	Did researchers adhere to the study protocol?
12	++	++	++	+	+	+	+	++	-	+
13	++	++	+	+	+	+	++	+	-	+
14	++	++	++	+	+	++	+	++	-	+
15	++	++	+	+	+	-	++	++	++	+
16	++	++	++	+	+	+	+	++	-	+
17	+	+	+	+	+	+	++	++	--	+
18	++	++	++	+	+	+	++	++	+	+
19	++	+	++	+	+	+	++	++	++	+
20	++	++	++	+	+	+	++	++	-	+
21	++	++	++	+	+	+	+	+	-	+

22	++	++	++	+	+	+	-	++	-	+
23	++	++	++	+	+	+	++	+	-	+
24	++	++	+	+	+	+	+	+	-	+
25	++	++	+	+	+	+	++	+	-	+
26	++	++	++	+	+	+	++	+	++	+
27	++	++	++	+	+	+	++	+	-	+
28	++	++	++	++	+	+	++	++	-	+
29	++	++	+	+	+	+	++	+	-	+
30	++	++	+	++	+	+	++	-	--	+
31	++	++	++	++	+	+	++	-	-	+
32	++	++	+	++	+	+	++	+	-	+
33	++	+	++	+	+	++	++	++	-	+
34	++	++	+	++	+	+	-	++	++	+
35	+	+	-	-	+	+	+	++	+	++
36	++	++	++	++	+	+	++	+	+	++
37	+	++	++	+	+	+	++	++	+	+

38	+	++	++	+	+	+	++	++	+	+
39	++	++	+	++	+	+	++	+	+	++
40	++	+	+	++	+	+	+	+	-	-
41	++	++	++	+	+	+	++	+	+	+
42	++	+	++	+	+	+	+	-	++	+
43	++	+	++	+	+	-	+	-	++	+
44	++	++	++	+	+	+	+	-	++	+
45	++	++	++	+	+	+	++	-	+	+
46	++	++	+	+	+	+	+	+	-	+
47	++	++	+	+	+	+	+	-	++	+
48	++	++	+	+	+	+	++	+	-	+
49	++	++	+	+	+	+	-	+	-	+
50	++	++	++	+	+	+	+	++	-	+
51	++	++	++	+	+	+	+	++	-	+
52	+	++	++	++	+	+	+	++	--	-
53	++	++	++	++	+	+	++	++	++	+

54	+	++	++	++	+	++	-	++	++	+
55	++	++	-	++	+	-	++	+	+	+
56	+	+	++	+	+	++	+	++	-	+
57	++	++	+	+	+	+	++	+	+	+
58	++	++	+	+	+	+	+	+	-	+
59	++	++	+	+	+	+	++	+	-	+
60	++	++	+	+	+	+	+	+	-	+
61	++	++	+	+	+	--	++	+	-	+
62	++	++	+	+	+	--	++	+	-	+
63	++	++	+	+	+	+	+	+	-	+
64	++	++	+	+	+	+	+	+	-	+
65	++	++	+	+	+	+	--	+	-	+
66	+	+	+	++	+	--	++	+	-	+
67	++	++	+	+	+	+	+	+	-	+
68	++	++	+	+	+	+	+	+	-	+
69	++	++	+	+	+	+	+	+	-	+

70	++	++	+	+	+	--	++	--	-	+

Abbreviations: Ref, reference number; ++, definitely low risk of bias; +, probably low risk of bias; -, probably high risk of bias; –, definitely high risk of bias.

Table S2: Frequency of assays used in extracted papers

Domain tested	Assay	Frequency of use
Cell viability/proliferation	MTT	29
	MTS	4
	CCK-8	2
	XTT	2
	TB staining	3
	WST-1	1
	CellTiter Blue	3
	CellTiter Glo	1
	PrestoBlue	1
	AlamarBlue	1
	LDH	1
	DAPI staining	1
	Toxilight	1
	SRB	1

	Calcein viability	1
	Rhodamine stain	2
	Crystal violet staining	1
	CyQUANT	1
	[3H]-thymidine	1
	Unspecified proliferation assay	4
	Unspecified toxicity assay	1
Cell migration	Scratch/wound healing	18
	Boyden chamber	6
	Ibidi culture insert assay	1
Apoptosis	Caspase-Glo 3/7	3
	Caspase-3	3
	Apo-one homogeneous caspase 3/7 assay	2
	TUNEL	7
	Annexin V/PI staining	9
	Comet	1

Other	ELISA	14
	Western blot	10
	SDS-PAGE	1
	HPLC	1
	LSC	1
	Von Kossa staining	1
	Alizarin red staining	1
	Amplex Red Peroxidase	1
	Bone nodule formation	1
	TEM cellular analysis	2
	Cell morphology analysis	3
	IHC staining	2
	Flow cytometry	9
	ALP	4
	BCA protein assay	3
	Smad-3 inhibition assay	1

Osteogenic differentiation	1
Immunofluorescence analysis	1
Senescence associated β galactosidase staining	1
TRAP staining	1
Resorption assay (Coomassie Brilliant Blue)	1
Masson’s trichrome staining	1
DCPMO	3
Osteocalcin synthesis	1
Immunostaining and confocal microscopy	1

Abbreviations: MTT, (4,5-dimethylthiazol-2-yl)-2,5-diphenyl-2H-tetrazolium bromide assay; MTS, 3-(4,5-dimethylthiazol-2-yl)-5-(3-carboxymethoxyphenyl)-2-(4-sulfophenyl)-2H-tetrazolium assay; CCK-8, Cell counting kit-8; XTT, - 2,3-Bis-(2-Methoxy-4-Nitro-5-Sulfophenyl)-2H-Tetrazolium-5-Carboxanilide

Assay; TB, trypan blue; WST-1, Water Soluble Tetrazolium Salts-1; LDH, Lactase Dehydrogenase; DAPI, 4′,6-diamidino-2-phenylindole; SRB, sulforhodamine B assay; TUNEL - Terminal deoxynucleotidyl transferase (TdT) dUTP Nick-End Labelling assay; Annexin V/PI staining, Annexin V/propidium iodide; ELISA, Enzyme-Linked Immunosorbent Assay; SDS-

PAGE, Sodium dodecyl-sulfate polyacrylamide gel electrophoresis; HPLC, High-performance liquid chromatography; LSC, Liquid Scintillation Counting; TEM, Transmission Electron Microscopy; IHC, Immunohistochemistry ; ALP, Alkaline phosphatase assay kit; BCA, Bicinchoninic acid; TRAP, Tartrate-resistant acid phosphatase; DCPMO, Direct Contrast-Phase Microscopic Observation.
